# Attitudes toward expanding nurses’ authority

**DOI:** 10.1186/s13584-015-0005-z

**Published:** 2015-09-01

**Authors:** Hana Kerzman, Dina Van Dijk, Limor Eizenberg, Rut Khaikin, Shoshi Phridman, Maya Siman-Tov, Shoshi Goldberg

**Affiliations:** Nursing Division, Chaim Sheba Medical Center, Tel-Hashomer, Israel; Ben-Gurion University of the Negev, Negev, Israel; Tel Aviv Sourasky Medical Center, Tel Aviv, Israel; Nursing Division, Tel Aviv University, Tel Aviv, Israel; Wolfson Medical Center, Holon, Israel; Gertner Institute for Epidemiology and Public Health Policy, Tel-Hashomer, Israel

**Keywords:** Attitudes, Expansion of nurses’ authority, Acute hospital, Job satisfaction, Workload, Burnout, Professional autonomy, Inter-professional cooperation

## Abstract

**Background:**

In recent years, an increasing number of care procedures previously under the physician’s authority have been placed in the hands of registered nurses. The purpose of this study was to examine the attitudes of nurses towards expanding nurses’ authority and the relationships between these attitudes and job satisfaction facets, professional characteristics, and demographics.

**Method:**

A cross-sectional study was conducted between 2010 and 2011 in three major medical centers in Israel. Participants included 833 nurses working in 89 departments. Attitudes toward the expansion of nurses’ authority were assessed by self-report questionnaire, as well as job satisfaction facets including perception of professional autonomy, nurse-physician working relations, workload and burnout, perceptions of quality of care, and nursing staff satisfaction at work.

**Results:**

Nurses reported positive attitudes toward the expansion of nurses’ authority and moderate attitudes for interpretation of diagnostic tests in selected situations. The results of multivariate regression analyses demonstrate that the nurses’ satisfaction from professional autonomy and work relations were the most influential factors in explaining their attitudes toward the expansion of nurses’ authority. In addition, professionally young nurses tend to be more positive regarding changes in nurses’ authority.

**Conclusions:**

In the Israeli reality of a nurse’s shortage, we are witnessing professional transitions toward expansion of the scope of nurses’ accountability and decision–making authority. The current research contributes to our understanding of attitudes toward the expansion of nurses’ authority among the nursing staffs. The findings indicate the necessity of redefining the scope of nursing practice within the current professional context.

## Introduction

In the wake of modern organizational and economic changes that have intensified the shortage of medical and nursing staff, the World Health Organization (WHO) has declared that the major aims of health systems everywhere include achieving an appropriate mix of medical care providers, while developing new roles for nurses working in the community as well as in acute care departments [1].

## Background

The debate regarding the expansion of the nurse’s role focuses on two core concepts: authority and responsibility. The delegation of *authority* from one professional to another, thus, implies transfer of the legal power to make decisions to a person formerly lacking in such power. Such professional authority represents one of the most important components in the growing professionalization of nursing [2,3]. In contrast, *responsibility* is defined as the obligation to provide an account of actions taken [4]. Creation of a balance between authority and responsibility underlies the drawing of professional boundaries.

Over the years, a wide gap has developed between nurses’ advanced practice and the professional authority which they have earned during their education. Advanced registered nurses practitioners in the community are therefore performing more than ever duties once reserved for physicians, such as prescribing medication, ordering tests and determining continuation of care [5-7].

With respect to the expansion of nurses’ authority in the community, the literature suggests that this trend has led to three important outcomes: Increased patient satisfaction, improved responsiveness to treatment and reduced re-hospitalizations [7-9].

Expansion of nurses’ authority is considered to be an important contributor to the creation of a working environment that encourages professional autonomy [10-12] while increasing satisfaction and reducing burnout and workload [5,13,14]. Research has also found that the higher the level of satisfaction and professional autonomy enjoyed by the nurses, the better the care they award their patients [15]. Moreover, level of satisfaction has been shown to be directly related to nurses’ intentions to leave their current place of employment [16,17].

Riva notes that *cooperation*, as a managerial strategy to replace hierarchical decision-making, has altered the distribution of authority that have maintained the boundaries between medicine and nursing as professions [18]. In recent years, physician-nurse relations have been changed. Relations rooted in rigid hierarchy have changed into relations based on cooperation and teamwork. One of the factors most influencing this process is the expansion of the nurses’ authority [19,20].

Nonetheless, the literature remains divided regarding the effect of the expansion of nurses’ authority on the quality of physician-nurse relations. According to Manias and Street, actions promoting expansion of nurses’ authority positively impact teamwork [21]. Alternatively, other researchers found that these same actions can introduce additional tensions into physician-nurse relations [22,23].

Several studies report positive attitudes held by physicians and nurses toward expansion of nurses’ authority in various areas, based on the belief that expansion improves quality of care [24-26]. Similar findings were obtained with regard to permitting nurses to write prescriptions for chronically ill patients [27]. In Israeli healthcare, positive attitudes were mostly found among younger and more educated nurses [26]. The majority of the respective studies were conducted among community-based physicians, with fewer conducted among nurses working in hospitals.

This paper is our attempt to examine Israeli nurses’ attitudes toward the expansion of nurses’ authority in acute care departments and the relationships between these attitudes and demographic variables, professional factors, and job satisfaction facets, such as professional autonomy, nurse-physician working relations, workload, burnout, quality of patient care and job satisfaction.

## Method

### Design

A cross-sectional study was conducted as part of a major research dealing with the effect of expanded authority on nurses’ professional development and on the quality of nursing care.

### Setting

The settings for this study were three large public medical centers in an urban area in the center of Israel. These medical centers provide high quality medical care at no cost to all patients regardless of their race/ethnicity, religious affiliation, or socio-economic status. Each of these centers has a wide range of medical departments (e.g., internal medicine, surgery, geriatric, oncological, pediatric units, acute care, delivery room, and others).

### Sample

A convenience sample of 833 nurses was recruited from the internal medicine, surgery, and geriatric departments (n = 89) at the above-mentioned three medical centers with a response rate of 53%. This rate was similar to those reported by others studies found in the literature [16,28,29].

### Data collection tools

The following research instruments were used to investigate the study variables:

*Attitudes toward expansion of nurses’ authority* were measured by a structured questionnaire available in the literature and adapted to the needs of the current research [26]. This measure included three domains: 1. The areas of expanding nurses’ authority in acute care assessed by a six-item. The internal reliability on Cronbach’s α was 0.88 in this study. 2. The potential influence of nurses’ expanded authority on quality improvement assessed by a three-item (e. g., health outcomes, economic efficiency and patient satisfaction). The Cronbach’s α was 0.85 in the current study. 3. The importance of expanding nurses’ authority was measured by a single-item question. All attitudes items’ scales were ranged on a six-point Likert Scale.

*Job satisfaction* included five facets:1. Professional autonomy was assessed using a previously validated questionnaire available in the literature and referring to nurses’ autonomy regarding decision making on the subject of patient care [30]. The questionnaire included 15 items with a Cronbach’s α = 0.82 in this study. 2. Nurse-physician work relations were measured using a six-item questionnaire, available in the literature, examining feelings regarding work relations and the level of cooperation between physicians and nurses [31]. Cronbach’s α of the instrument was 0.73 in this study. 3. Workload & Burnout were measured using the SMBM (Shirom–Melamed; Burnout Measure) [32]. The questionnaire includes 14 items with Cronbach’s α = 0.93 in this study. 4. Quality of care was measured by a targeted questionnaire particularly constructed by the research team for this study. The questionnaire referred to different aspects of perception of quality of care, such as patient safety, patient privacy, patient education, and response to patient needs. The questionnaire included 13 items with high internal reliability (Cronbach’s α = 0.93). 5. *Job satisfaction* was assessed by a questionnaire especially constructed by the research team for this study. The questionnaire includes 10 items related to different aspects of nurses’ satisfaction with their work (Cronbach’s α = 0.79). All job satisfaction facets’ scales were ranged on a five-point Likert Scale, except for the workload & burnout measure which ranged on a seven-point Likert Scale.

Additionally, respondents were asked to provide data on their *demographic and professional characteristics* (age, gender, professional seniority and type of employment (part- or full-time job), academic accreditation and management position).

A pre-test study was performed (n = 20) for evaluation of the data collection process, questionnaire length, and comprehensibility. The participants were asked to provide feedback regarding clarity and readability of the instruments and found it to be clear and concise.

The Ethics Committee approval was obtained from each of the medical centers. The participants were included after oral and written informed consents had been obtained.

Data were collected between June 2010 and July 2011.

### Statistical analysis

The demographic and professional characteristics and job satisfaction facets were descriptively analyzed using frequencies, percentages, and measures of central tendency (i.e., means and medians), and measures of variability (i.e., standard deviation and ranges) based on measurement scale. Univariate analyses (e.g., Correlations and t-tests) were used to test the relationships between demographics and job satisfaction facets to the attitudes toward expanding nurses’ authority.

Finally, three models of multiple linear regressions were used to predict the three domains of attitudes toward the expanding nurses’ authority, by demographic and professional characteristics (gender, academic accreditation, management position and professional seniority) and job satisfaction facets (job satisfaction, professional autonomy, work relations, workload & burnout and quality of care) that were found to be significant in the univariate analyses. A value of p < 0.05 was considered to be statistically significant. Data analysis was performed using SPSS software, version 19 (SPSS. Inc. Chicago IL).

## Results

### Sample characteristics

The majority of the sample were women (n = 675; 81.4%), average age 38.2 (*SD* = 9.5), average seniority at the current job 10.1 (*SD* = 13.1) years. The majority (n = 640; 76.8%) held academic accreditation; about 52% (n = 432) had completed post basic education. Seventy percent (n = 585) held full-time jobs and 22.5% (n = 188) held management positions.

### Attitudes toward expanding nurses’ authority

Table 1 describes the three domains of attitudes toward expanding nurses’ authority and the means and SDs for each item within the three domains. The highest positive attitudes (on a five-point scale) were regarding the *importance* of expanding nurses’ authority (M = 4.8, SD = 1.2). This means that the nurses are highly evaluate the importance of the expansion of their authority. Additionally, within the domain of areas of expansion, the area of *treatment decision* received the highest positive rating (M = 4.7; SD = 3.1), whereas the areas of *diagnosis* received the lowest ratings: specifically, referral to diagnostic tests and diagnosis in selected situations, received the lowest positive ratings (M = 3.8, SD = 1.5; M = 3.6, SD = 1.5; respectively). These results mean that nurses highly believe that nurses’ authority should include treatment decision, but are less positive about expanding nurses’ authority to include diagnoses.

**Table 1:** **Means and SDs of the nurse’s attitudes regarding expanding nurses’ authority in three domains: 1. Areas of expanding 2. Quality improvement 3. Importance of expending**

	**Mean (SD)**
**1. Areas of expansion**	
Medical history/ physical assessment	4.1 (1.5)
Prescription privileges	4.1 (1.5)
Treatment decisions	4.7 (3.1)
Referrals to lab tests and their interpretation	4.3 (1.4)
Referrals to diagnostic tests	3.8 (1.5)
Diagnosis in selected situations	3.6 (1.5)
**2. Quality improvement**	
Improvement health outcome	4.1 (1.1)
Greater cost-effectiveness	4.2 (1.4)
Increase of patient satisfaction	4.5 (1.2)
**3. Importance of expanding**	4.8 (1.2)

Note: the attitudes are measured on a 5-point scale.

Among the domain of quality improvement, the *increase of patient satisfaction* received the highest positive rating (M = 4.5; SD = 1.2), meaning that the nurses highly believe that expanding their authority will improve patients satisfaction.

### Demographic and professional characteristics and attitudes toward expanding nurses’ authority

Table 2 presents factors which might influence attitudes toward expanding nurses’ authority. A significant difference was found between male and female nurses in areas of expanding authority (M = 4.3; SD = 1.1 versus M = 4.0; SD = 1.2, p < .01), quality improvement (M = 4.6; SD = 1.1 versus M = 4.4; SD = 1.1, p < .05) and importance of expanding authority (M = 5.1; SD = 1.1 versus M = 4.8; SD = 1.2, p < .05). Male nurses reported more positive attitudes than female nurses regarding expansion of authority in these domains.

**Table 2:** **Mean differences of attitudes toward expanding nurses’ authority (three domains) by demographic and professional characteristics**

	**1. Areas of expanding**	**2. Quality improvement**	**3. Importance of expanding**
	**Mean (SD)**	**Mean (SD)**	**Mean (SD)**
**Gender**			
Male	4.3 (1.1)	4.6 (1.1)	5.1 (1.1)
Female	4.0 (1.2)	4.4 (1.1)	4.8 (1.2)
	t = −2.8**	t = −2.1*	t = −2.1*
**Academic Accreditation**			
No	3.9 (1.3)	4.5 (1.1)	4.7 (1.3)
Yes	4.1 (1.2)	4.5 (1.1)	4.9 (1.1)
	t = −1.8(NS)	t = −.17(NS)	t = −1.9*
**Management Position**			
No	4.1 (1.2)	4.4 (1.1)	4.8 (1.1)
Yes	4.4 (1.1)	4.6 (1.1)	4.9 (1.1)
	t = −3.9**	t = −3.9*	t = −1.8(NS)

*p-value < .05 **p-value < .01.

Table 2 also shows that academically accredited nurses reported more positive attitudes toward importance of expanding nurses’ authority, in comparison to nurses without academic accreditation (M = 4.9; SD = 1.1 versus M = 4.7; SD = 1.3, p < .05). The higher level of an academic degree the more positive the attitudes that were reported by nurses. In addition, nurses occupying management positions reported significantly higher positive attitudes toward areas of expanding (M = 4.4; SD = 1.1 versus M = 4.1; SD = 1.2, p < .01) and quality improvement in comparison to nurses without management positions (M = 4.6, SD = 1.1 versus M = 4.4, SD = 1.1, p < .05).

Surprisingly, no significant differences were found between academically accredited nurses and nurses without academic accreditation in areas of expanding (M = 4.1; SD = 1.2 versus M = 3.9; SD = 1.3, p > .05) and quality improvement (M = 4.5; SD = 1.1 versus M = 4.5; SD = 1.1, p > .05). Additionally, no significant differences were found between nurses holding management position and nurses without management position concerning to importance of expanding (M = 4.9; SD = 1.1 versus M = 4.8; SD = 1.1, p > .05) indicating that all nurses regardless to their position have high positive attitudes regarding importance of expanding.

As can be seen in Table 3, age and professional seniority were negatively correlated with attitudes regarding the importance of expanding (r = −.048, p > .05 and r = −.11, p < .001 consequently); meaning that the younger and less experienced reported more positive attitudes. In addition, types of employment was positively correlated with attitudes toward areas of expanding (r = .074, p < .05), meaning that nurses working in full time job reported more positive attitudes than nurses working part time job.

**Table 3:** **Pearson correlations between attitudes toward expanding nurses’ authority (three domains), demographic and professional characteristics and Job Satisfaction Facets**

**Attitudes:**	**1. Areas of expanding**	**2. Quality improvement**	**3. Importance of expanding**
**Demographics**			
Age	-.048	-.017	-.133**
Professional seniority	-.111**	-.059	-.109**
Types of employment	.074*	.047	.024
**Job Satisfaction Facets**			
Job Satisfaction	.188**	.192**	.178**
Work Relations	.221**	.236**	.253**
Professional Autonomy	.213**	.224**	.210**
Workload & Burnout	-.070*	-.116**	-.094**
Quality of Care	.141**	.207**	.159**

*p-value < .05 **p-value < .01.

### Job satisfaction facets and attitudes toward expanding nurses’ authority

All job satisfaction facets were significantly correlated with attitudes toward expanding nurses’ authority (p < .001). It was found that the greater the nurses’ job satisfaction, work relations, professional autonomy and perceptions of quality of care – the more positive their attitudes toward expanding nurses’ authority. In addition, the less workload and burnout, the more positive attitudes of nurses toward expansion their authority (r = −.07, p < .05) (Table 3).

In order to test what are the most influential factors affecting the nurses’ attitudes toward expansion of their authorities; we conducted three multiple regression models (see Table 4), one for each domain of expanding authority (i.e., area of expanding authority, quality improvement, importance of expanding authority). Each of the model contained the variables that were found to be significant in the univariate analyses - as independent factors. Since our data was obtained from three hospitals, we controlled for the type of hospital by computing two dummy variables and entering them at the first step of each regression model. The effects of the dummy variables were non-significant indicating that type of hospital did not affect the attitudes toward expanding nurses’ authority.

**Table 4:** **Results of three models of linear regression to predict attitudes toward expanding nurses’ authority (three domains), by demographic and professional characteristics and Job Satisfaction Facets**

**Attitudes:**	**1. Areas of expanding**			**2. Quality improvement**			**3. Importance of expanding**		
	**beta**	**t**	**sig**	**beta**	**t**	**sig**	**beta**	**t**	**sig**
***Demographic and professional characteristics***									
Gender	.07	1.83	NS	.06	1.52	NS	.08	2.07	*
Academic Accreditation (0-no 1-yes)	.03	.67	NS	-.03	-.80	NS	.05	1.27	NS
Management Position (0-no 1- yes)	.05	1.27	NS	-.04	-.90	NS	-.03	-.83	NS
Professional Seniority	-.09	−2.05	*	-.09	−2.24	*	-.10	−2.47	*
Job Satisfaction	.05	1.05	NS	.01	.25	NS	-.01	-.09	NS
Professional Autonomy	.14	3.07	**	.16	3.56	**	.13	2.70	**
Work relations	.14	3.22	**	.14	3.15	**	.18	4.20	**
Workload & Burnout	.02	.46	NS	-.01	-.29	NS	-.03	-.68	NS
Quality of Care	.01	.28	NS	.09	2.00	*	.05	1.04	NS

*p-value < .05 **p-value < .01.

The results of the three regression models are summarized in Table 4. The results of the regression analyses demonstrate that among the job satisfaction facets - the nurses’ perceptions of *professional autonomy* and *work relations* were the most influential factors in explaining nurses’ attitudes toward the expansion of their authority Specifically, the greater the nurses’ perceptions of professional autonomy and work relations the greater their willingness to expand the nurses’ authority. These two factors were consistently significant for all three domains of attitudes. Also, job satisfaction from *quality of care* was significantly predicted attitudes towards expansion, but only for one domain – quality improvement. Meaning that the more the nurse is satisfied with the quality of care the more positive attitudes she hold toward expansion of nurse’s authority, in terms of quality improvement.

Among the demographic and professional characteristics, only *professional seniority* found to be consistently related to the nurses’ attitudes (in all three domains); specifically, the senior the nurse the less positive attitudes he or she holds. This indicates that (professionally) young nurses tend to be more positive regarding changes in nurses’ authority, while the senior ones are less willing to welcoming these changes. Also, *gender* was a predictor of one domain of attitudes – importance of expanding of nurses’ authority, meaning that men are more positively viewing the importance of expansion authority than women.

## Discussion

Findings from the current research clearly indicate that nurses expressed positive attitudes toward the expansion of nurses’ authority as previously found in several studies [24,26,33]. Similarly, positive attitudes toward expanding of nurses’ authority were also obtained by Riva in what she considers to be “grey areas” and “exceptional cases” [18]. We suggest that these findings can be explained by the fact that nurses view the expansion of their authority as a positive development, one enabling them to exercise professional autonomy and establish more direct relations with patients while contributing to their ability to respond to their needs. In addition, study conducted in Israel showed a positive attitude towards expanding the authority of nurses among patients [34].

As to actions directly impacting medical care, such as referrals to diagnostic tests and their interpretation, the current research revealed that nurses were less supportive with respect to the expansion of nurses' authority in these domains, supporting Brodsky and Van Dijk findings [26]. However, the strength of these positive attitudes increased in reference to actions regarding accepted nursing practice, such as continuation of care and prevention of complications. Interestingly, it appears that this pattern of attitudes is similar to the attitudes held by physicians, as was shown in previous literature. Specifically, physicians are supportive in expanding nurses’ authority in domains of laboratory tests, care management for the chronically ill, writing of prescriptions, patients education and continuous care coordination, but not in domains which have strong impact on care and recuperation, such as invasive procedures, diagnosis, and advanced procedures [33,35-37].

Turning to nurses, we suggest that they are more reticent about accepting authority in the domain of diagnosis which is accompanied by greater responsibility for the provision of care.

### Factors relating to attitudes toward nurses’ expansion of authority

The relationships found between demographics and professional variables and the nurses’ attitudes toward expanding nurses’ authority indicated that gender and professional seniority were the most influential variables: specifically, male and professionally young nurses reported more positive attitudes toward expansion (though gender was related only to the importance of the expanding but not to the other two domains of expansion). These findings comply with those previously obtained by Brodsky and Van Dijk [26] and Riva [38].

Academically accredited nurses and nurses holding managerial positions expressed more positive attitudes toward this process than did nurses lacking academic accreditation or holding no managerial positions; however these two factors become non-significant in the multivariate analyses, indicating that they are less influential compared to professional seniority and gender. These results contrasted to the results of the previous studies who found stronger effects of academic accreditation and management position on attitudes toward expanding nurses authority [26,39].

The most significant factors that were found to be related to attitudes toward nurses’ expansion of authority were the three facets of job satisfaction: professional autonomy and physician-nurse work relations (which was found to be significant for all three attitudes domains), and quality of care (which was found to be significant only for the domain of quality improvement); while the other facets (workload & burnout and job satisfaction) were significant in the univariate analyses but not in the multivariate analyses. Manias and Street as well as Dahamsha and Golander have reported similar findings [21,40]. Also, a research conducted among nurses having the authority to write prescriptions found that the nurses reported an increase in their level of professional autonomy at work [12]. Other research, conducted among nurses working in hospitalization departments, found that with regards to work according to authority - expanding protocols likewise led to an increase in the level of the nurses’ professional autonomy [11].

### Study limitations

The study was conducted in three major medical centers located in Israel’s central region. It follows that the findings and conclusions must be taken as pertaining only to this geographic area and do not necessarily reflect the attitudes of physicians and nurses working in other medical facilities, especially in the peripheral areas of the country. Further research is needed to confirm the generalizability of our findings.

Additionally, nurses may have been influenced by the title of the research and may have responded to the questionnaires in the manner perceived to be more socially desirable.

Last, the study was a cross-sectional study with a ‘one time’ data collection and therefore cannot infer causal relationships between variables, but only correlational relationships.

### Relevance to clinical practice

Health systems currently find themselves pressured to change in response to rapid technological developments. Also to be considered are factors originating in other spheres, such as increasing cost-consciousness and a rapidly growing elderly population, both of which are contributing to demands for the expansion of community care, the rising number of patients hospitalized in acute-care department, and the consequent growing shortages of medical and nursing staff. Demands for new skills and new services are therefore rising.

The expansion of nurses’ authority thus appears called for as part of the response to these organizational and economic transformations, especially if we are to maintain high levels of patient care while containing costs.

To achieve these objectives we must go beyond recognition of the need to reallocate decision-making authority and reorganize work. We hope that such a reevaluation will promote awareness of the need to adopt such a policy in Israel. We also hope that the expansion of nurses’ authority will motivate a greater number of talented individuals to join the profession.

## Conclusion

The current research contributes to our understanding of attitudes toward the expansion of nurses’ authority among the nursing staffs in hospitalization departments in three medical centers in Israel.

The findings reported here contribute to broadening our understanding of how nurses perceive the outcomes of expanding nurses’ authority. They concur with respect to the anticipated outcomes of this policy shift, which they see as improvement in health outcomes and increased patient satisfaction. The nurses participating in the research tended to support such a decision and were generally convinced that it will engender positive improvements in teamwork together with increased nurse autonomy, improved patient care and greater satisfaction, changes that will reduce nurses’ workload and burnout.

Furthermore, based on these and other findings, we suggest that research should be conducted about patients’ attitudes toward this change in policy in the various departments maintained in hospitals throughout Israel.
